# Supplementary material for: A yeast phenomic model for the influence of Warburg metabolism on genetic buffering of doxorubicin
Source: Cancer Metab. 2019 Oct 23;7:9. doi: 10.1186/s40170-019-0201-3 (PMC6806529; doi:10.1186/s40170-019-0201-3)
Supplement: Supplementary file 9 — Additional file 9. GO term-specific heatmaps for REMc/GTF-enriched clusters. GO term-specific heatmaps for significant GO process terms were generated as described in methods and Figs. 3 and 4. Any related child terms are presented in subsequent pages of the parent file name. GO terms with more than 100 children, with 2 or fewer genes annotated to the term, or a file size over 300KB are not shown. All heatmaps are generated with the same layout (see Figs. 3 and 4). [file 40170_2019_201_MOESM9_ESM.bz2 › Additional_File9_GOTermHeatmaps/Additional_File9_GOTermHeatmap/transcription,_DNA-templated.pdf]

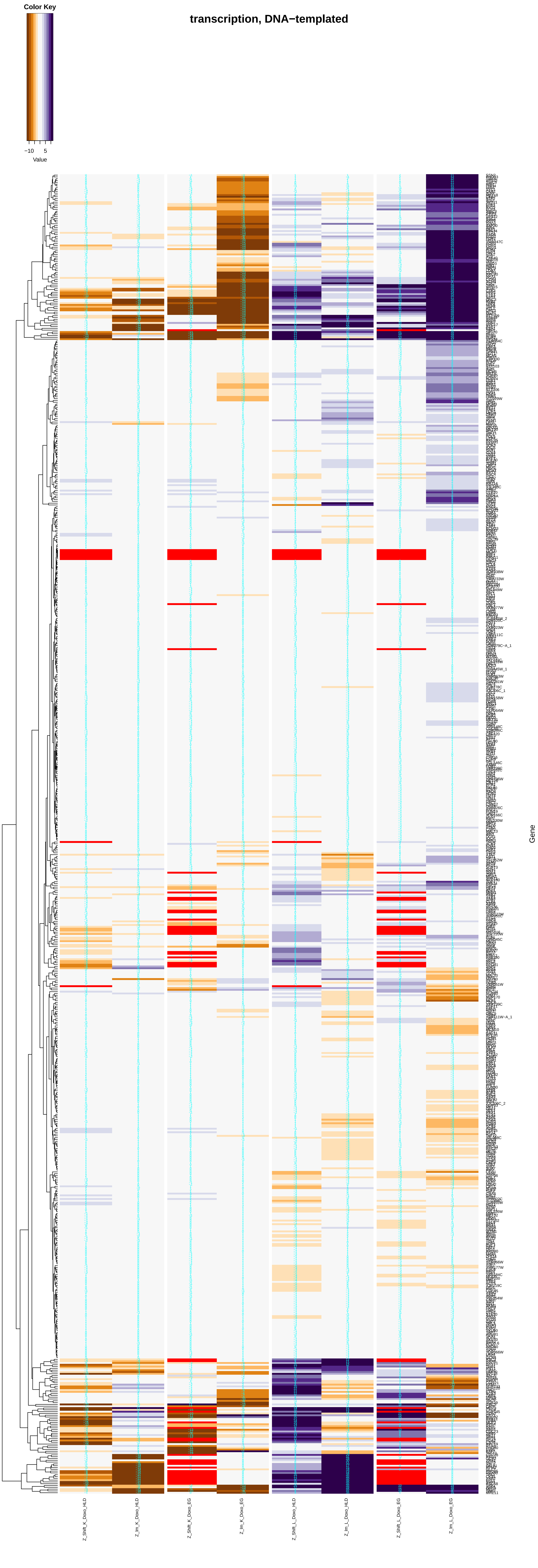
transcription, DNA−templated
Color Key
−10
5
Value
Gene
Z_Shift_K_Doxo_HLD
Z_lm_K_Doxo_HLD
Z_Shift_K_Doxo_EG
Z_lm_K_Doxo_EG
Z_Shift_L_Doxo_HLD
Z_lm_L_Doxo_HLD
Z_Shift_L_Doxo_EG
Z_lm_L_Doxo_EG

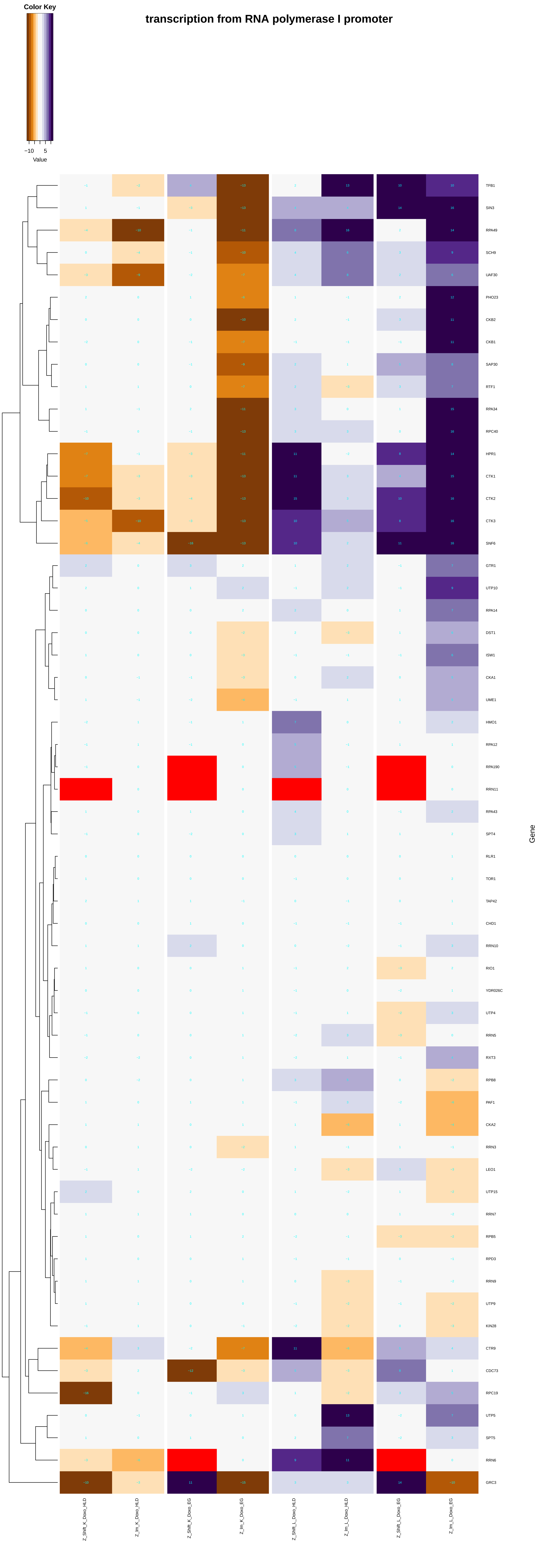

Color Key
−10
5
Value
transcription from RNA polymerase I promoter
TFB1
SIN3
RPA49
SCH9
UAF30
PHO23
CKB2
CKB1
SAP30
RTF1
RPA34
RPC40
HPR1
CTK1
CTK2
CTK3
SNF6
GTR1
UTP10
RPA14
DST1
ISW1
CKA1
UME1
HMO1
RPA12
RPA190
RRN11
RPA43
SPT4
RLR1
TOR1
TAP42
CHD1
RRN10
RIO1
YDR026C
UTP4
RRN5
RXT3
RPB8
PAF1
CKA2
RRN3
LEO1
UTP15
RRN7
RPB5
RPD3
RRN9
UTP9
KIN28
CTR9
CDC73
RPC19
UTP5
SPT5
RRN6
GRC3
Gene
Z_Shift_K_Doxo_HLD
Z_Im_K_Doxo_HLD
Z_Shift_K_Doxo_EG
Z_Im_K_Doxo_EG
Z_Shift_L_Doxo_HLD
Z_Im_L_Doxo_HLD
Z_Shift_L_Doxo_EG
Z_Im_L_Doxo_EG

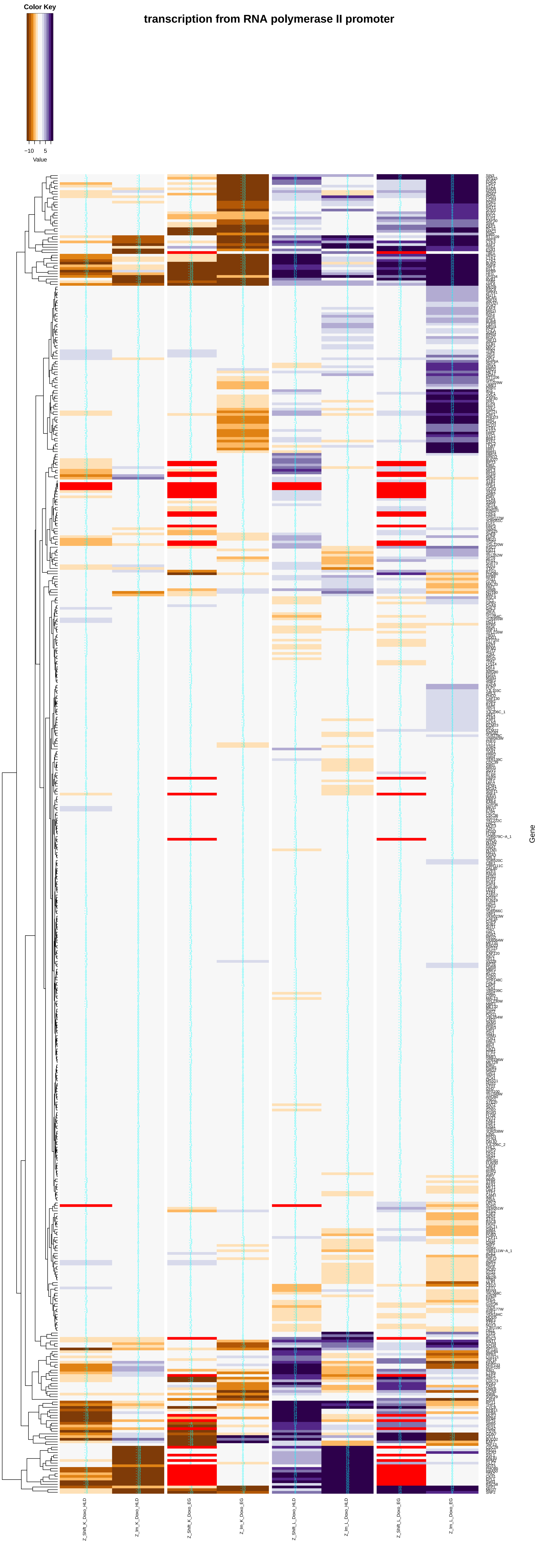

transcription from RNA polymerase II promoter
Color Key
−10
5
Value
Gene
Z_Shift_K_Doxo_HLD
Z_Im_K_Doxo_HLD
Z_Shift_K_Doxo_EG
Z_Im_K_Doxo_EG
Z_Shift_L_Doxo_HLD
Z_Im_L_Doxo_HLD
Z_Shift_L_Doxo_EG
Z_Im_L_Doxo_EG

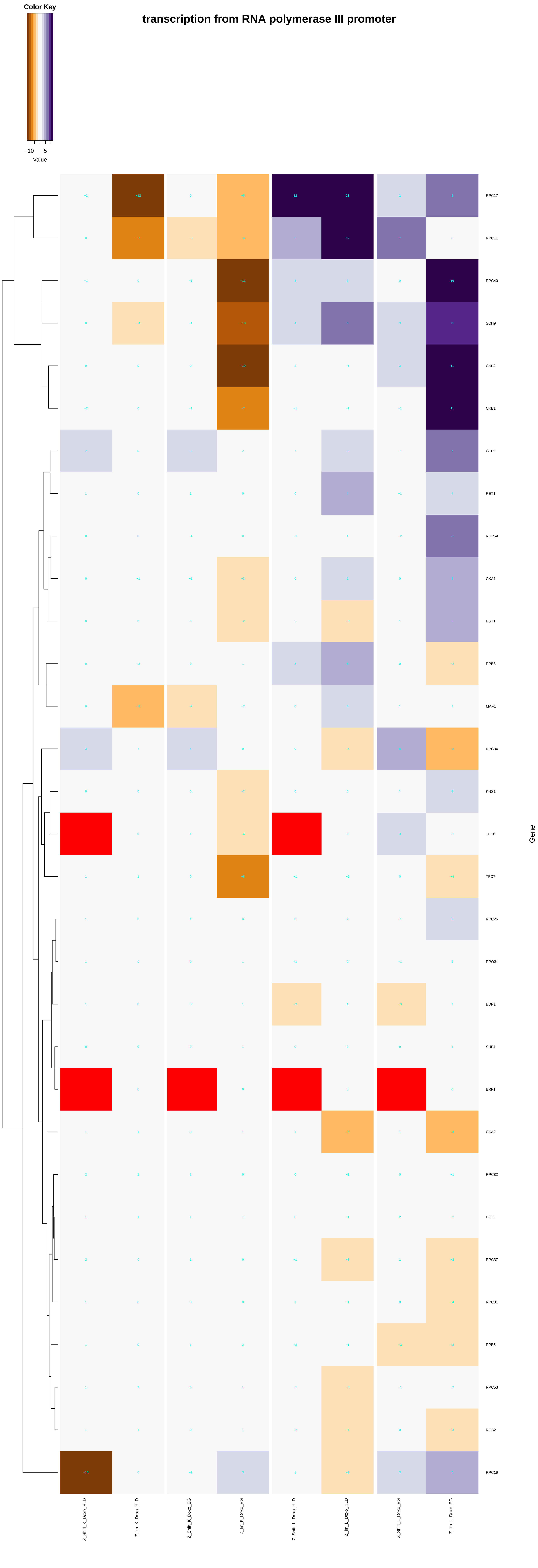
transcription from RNA polymerase III promoter
Color Key
−10
5
Value
RPC17
RPC11
RPC40
SCH9
CKB2
CKB1
GTR1
RET1
NHP6A
CKA1
DST1
RPB8
MAF1
RPC34
KNS1
TFC6
TFC7
RPC25
RPO31
BDP1
SUB1
BRF1
CKA2
RPC82
PZF1
RPC37
RPC31
RPB5
RPC53
NCB2
RPC19
Gene
Z_Shift_K_Doxo_HLD
Z_lm_K_Doxo_HLD
Z_Shift_K_Doxo_EG
Z_lm_K_Doxo_EG
Z_Shift_L_Doxo_HLD
Z_lm_L_Doxo_HLD
Z_Shift_L_Doxo_EG
Z_lm_L_Doxo_EG

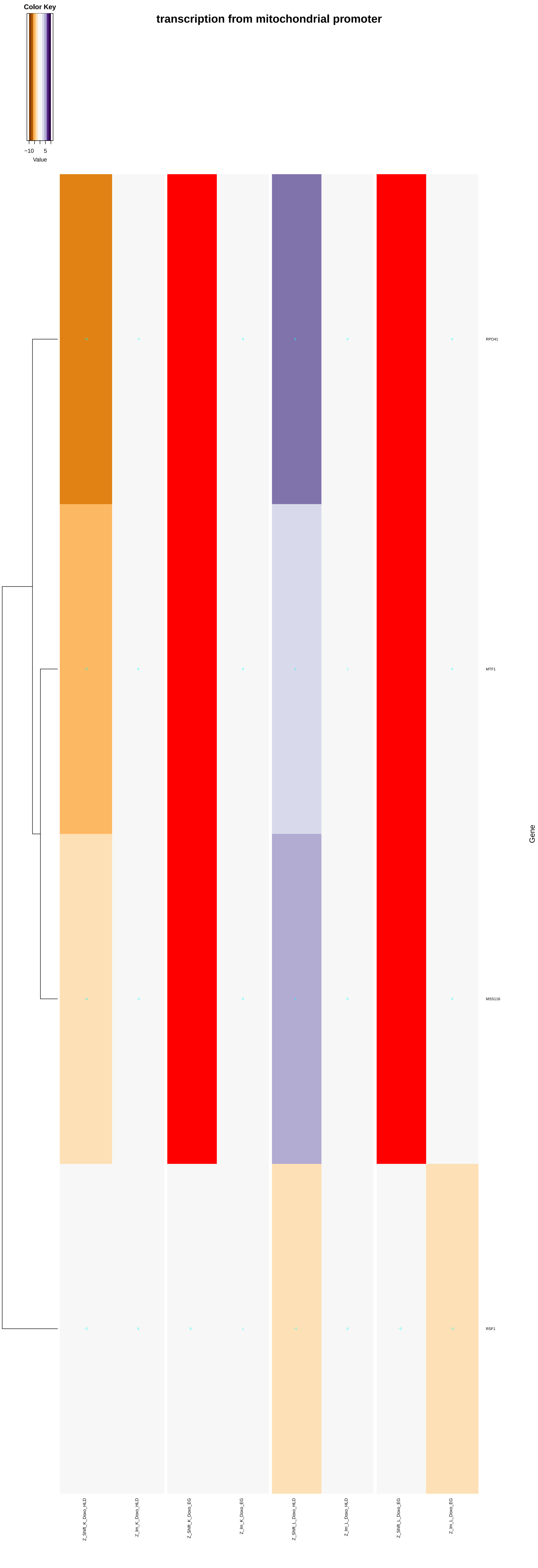

Color Key
−10
5
Value
transcription from mitochondrial promoter
RPO41
MTF1
MSS116
RSF1
Gene
Z_Shift_K_Doxo_HLD
Z_Im_K_Doxo_HLD
Z_Shift_K_Doxo_EG
Z_Im_K_Doxo_EG
Z_Shift_L_Doxo_HLD
Z_Im_L_Doxo_HLD
Z_Shift_L_Doxo_EG
Z_Im_L_Doxo_EG

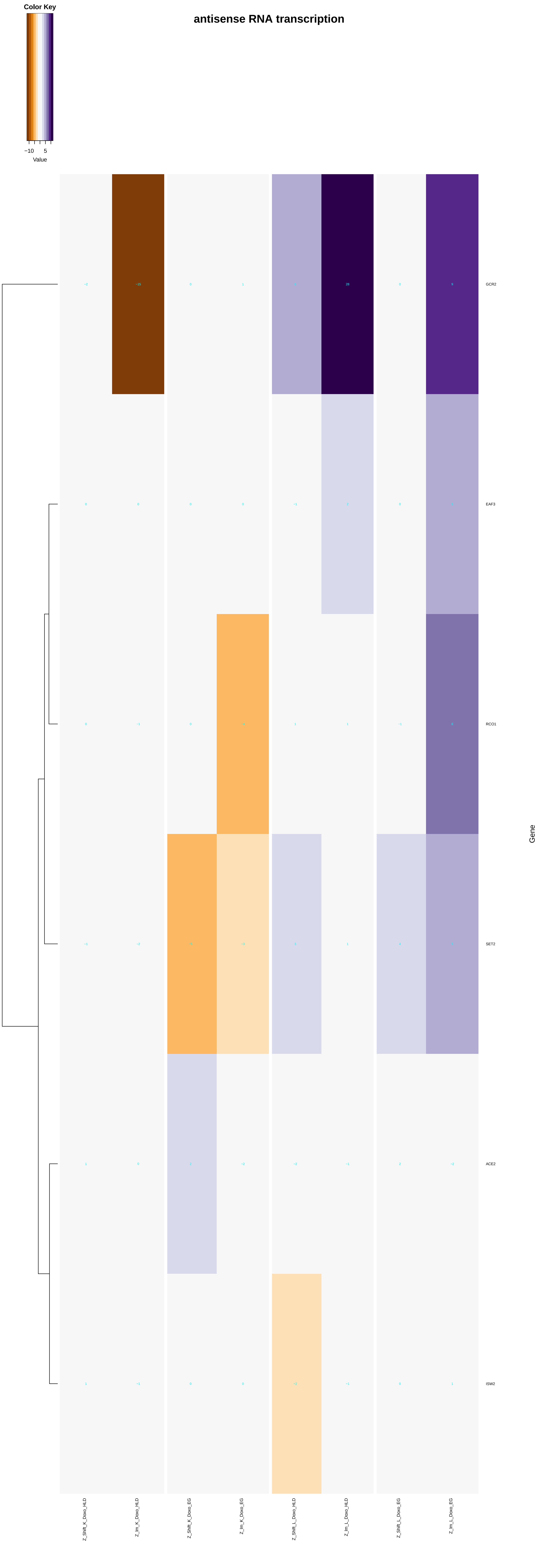
antisense RNA transcription
Color Key
−10
5
Value
GCR2
EAF3
RCO1
SET2
ACE2
ISW2
Gene
Z_Shift_K_Doxo_HLD
Z_Im_K_Doxo_HLD
Z_Shift_K_Doxo_EG
Z_Im_K_Doxo_EG
Z_Shift_L_Doxo_HLD
Z_Im_L_Doxo_HLD
Z_Shift_L_Doxo_EG
Z_Im_L_Doxo_EG

ncRNA transcription

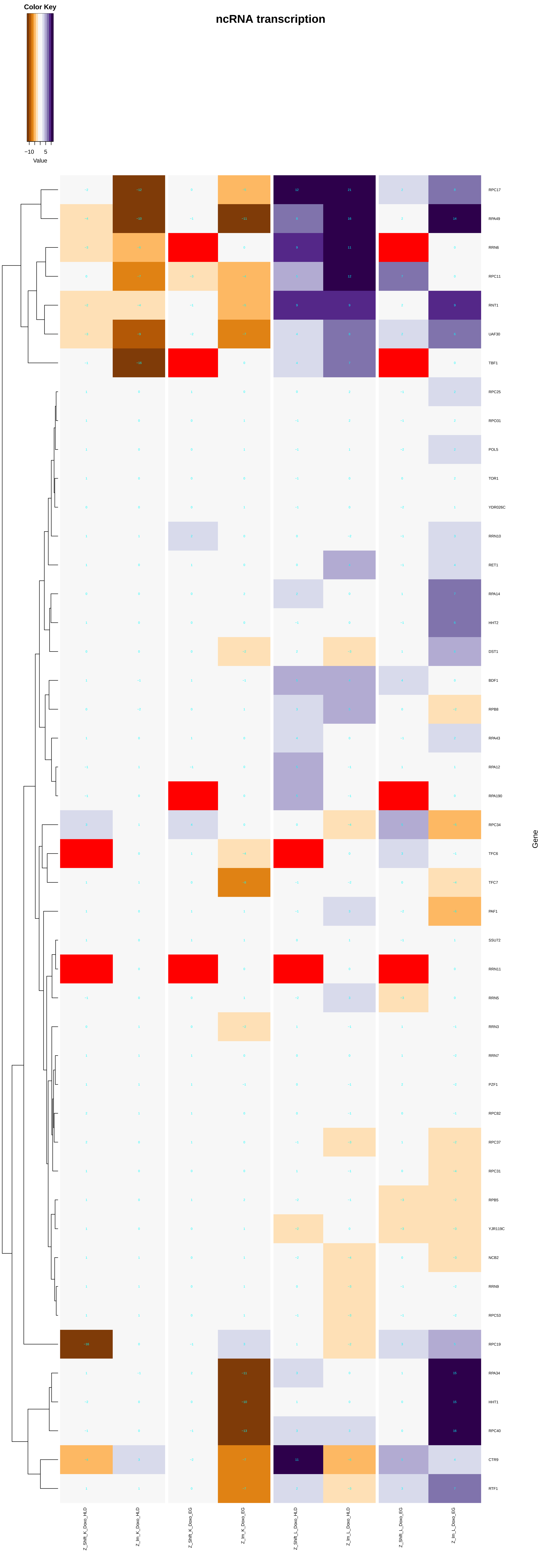

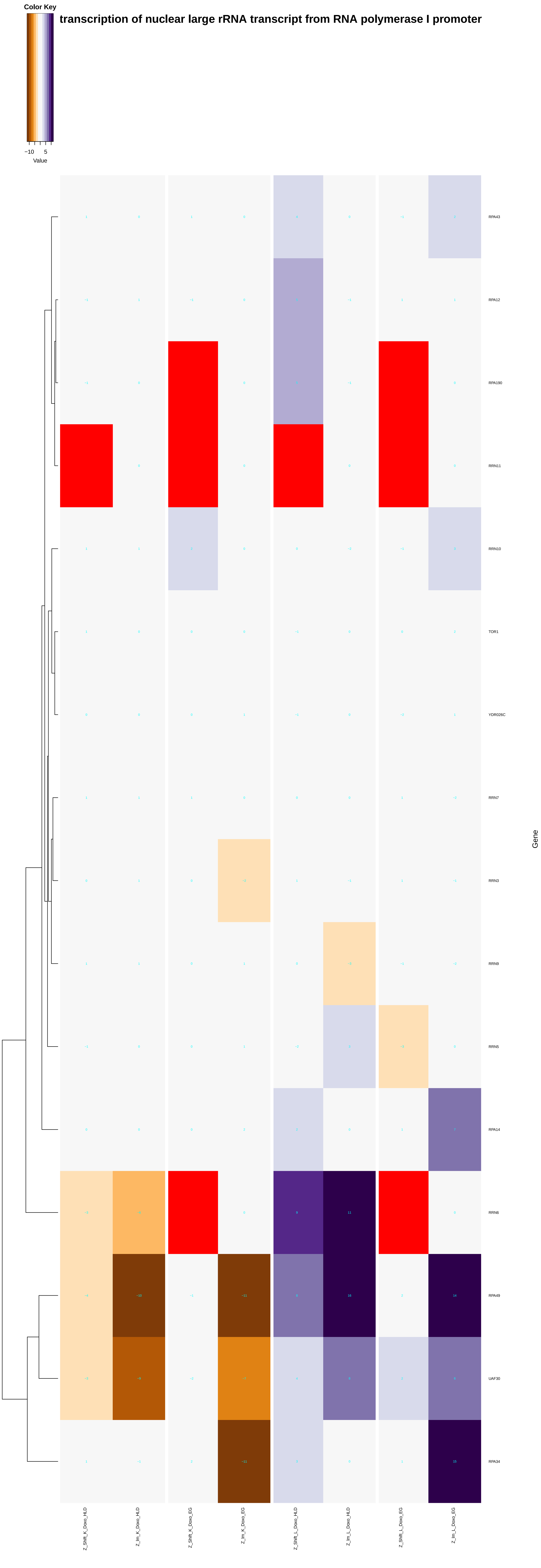
Color Key
−10
5
Value
transcription of nuclear large rRNA transcript from RNA polymerase I promoter
RPA43
RPA12
RPA190
RRN11
RRN10
TOR1
YDR026C
RRN7
RRN3
RRN9
RRN5
RPA14
RRN6
RPA49
UAF30
RPA34
Gene
Z_Shift_K_Doxo_HLD
Z_Im_K_Doxo_HLD
Z_Shift_K_Doxo_EG
Z_Im_K_Doxo_EG
Z_Shift_L_Doxo_HLD
Z_Im_L_Doxo_HLD
Z_Shift_L_Doxo_EG
Z_Im_L_Doxo_EG

snoRNA transcription from an RNA polymerase II promoter

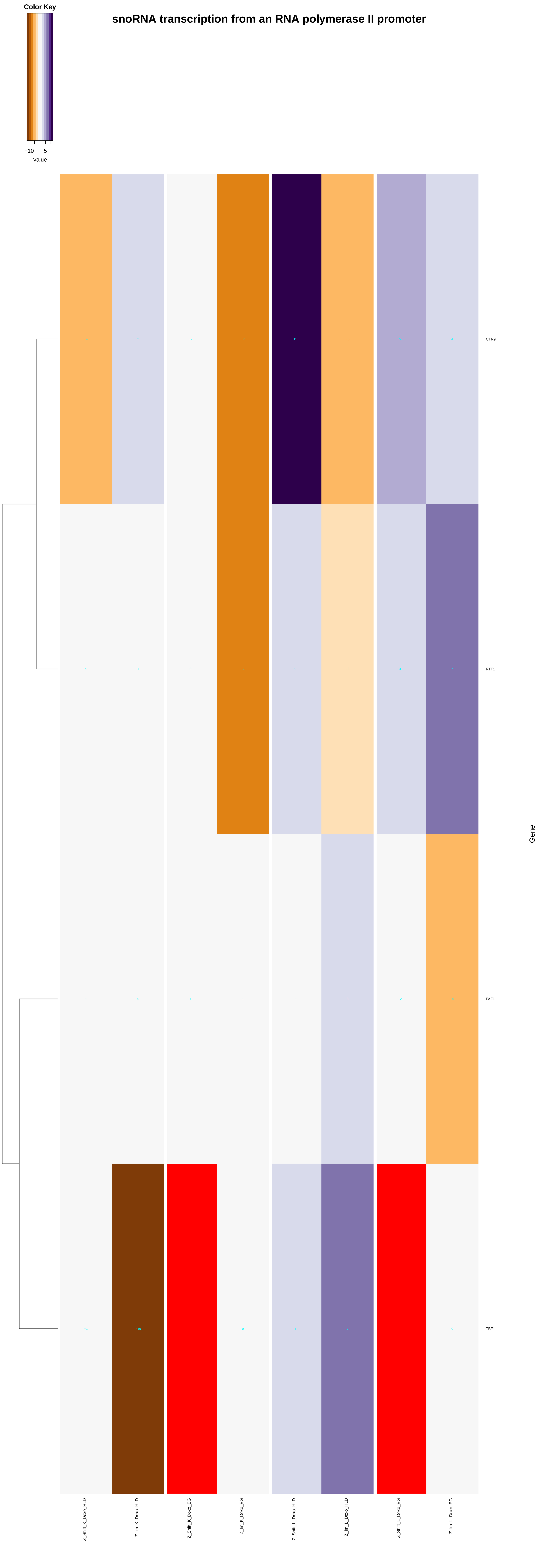

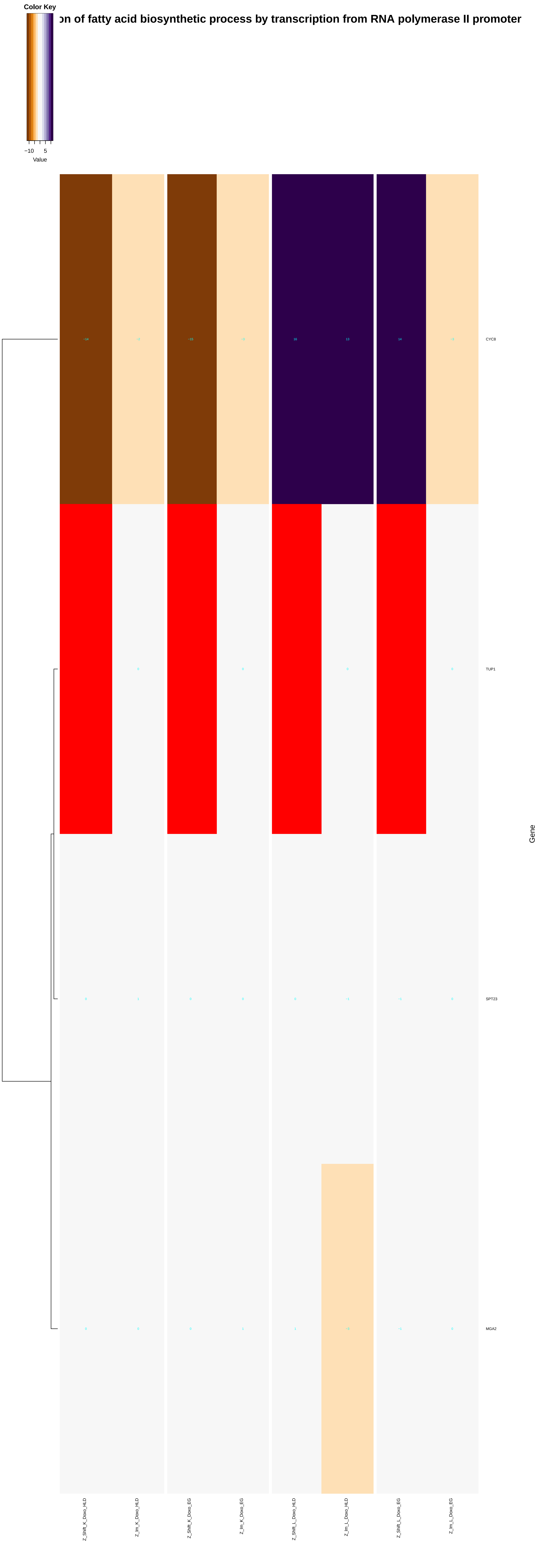

Color Key
−10
5
Value
on of fatty acid biosynthetic process by transcription from RNA polymerase II promoter
−14
−2
−15
−3
16
13
14
−3
CYC8
0
0
0
0
TUP1
0
1
0
0
0
−1
−1
0
SPT23
0
0
0
1
1
−3
−1
0
MGA2
Gene
Z_Shift_K_Doxo_HLD
Z_Im_K_Doxo_HLD
Z_Shift_K_Doxo_EG
Z_Im_K_Doxo_EG
Z_Shift_L_Doxo_HLD
Z_Im_L_Doxo_HLD
Z_Shift_L_Doxo_EG
Z_Im_L_Doxo_EG

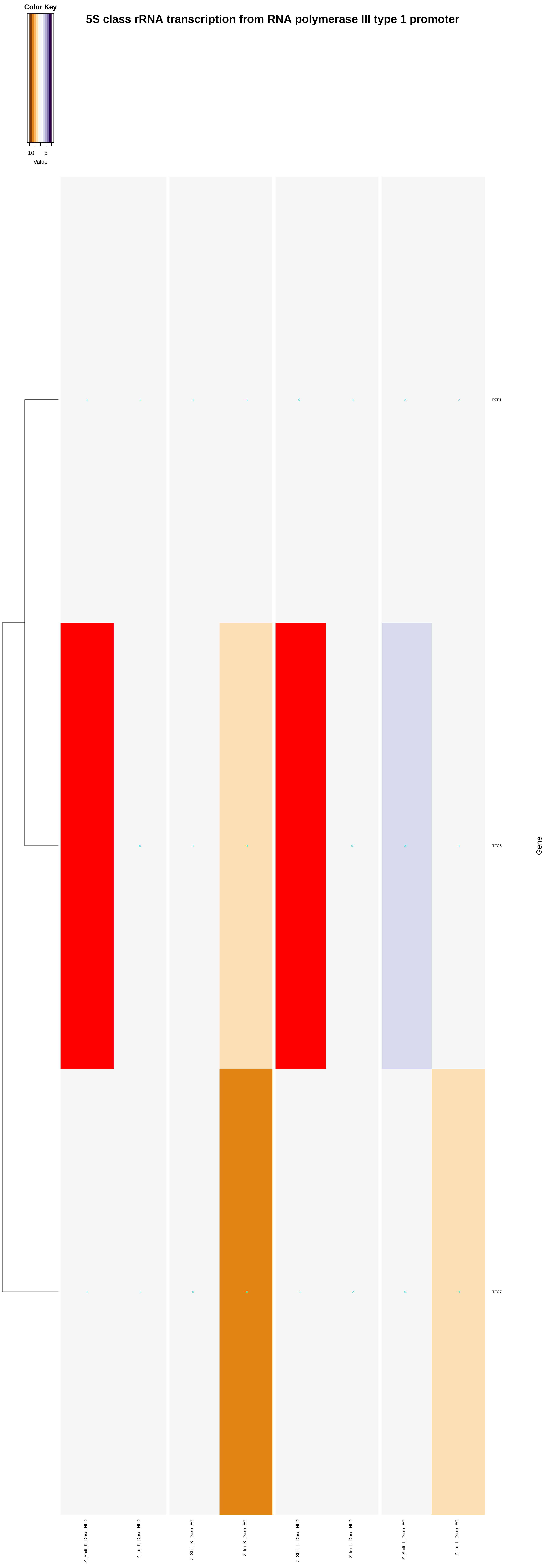
Color Key
−10
5
Value
5S class rRNA transcription from RNA polymerase III type 1 promoter
PZF1
TFC6
TFC7
Gene
Z_Shift_K_Doxo_HLD
Z_Im_K_Doxo_HLD
Z_Shift_K_Doxo_EG
Z_Im_K_Doxo_EG
Z_Shift_L_Doxo_HLD
Z_Im_L_Doxo_HLD
Z_Shift_L_Doxo_EG
Z_Im_L_Doxo_EG

tRNA transcription from RNA polymerase III promoter

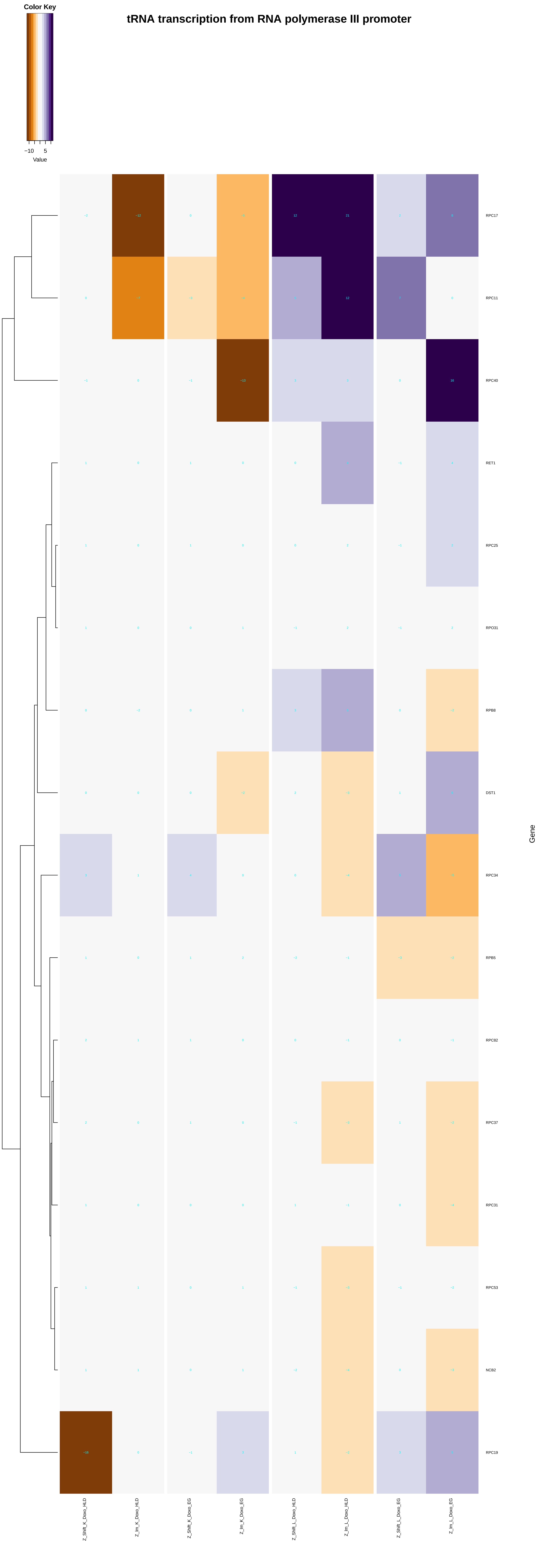

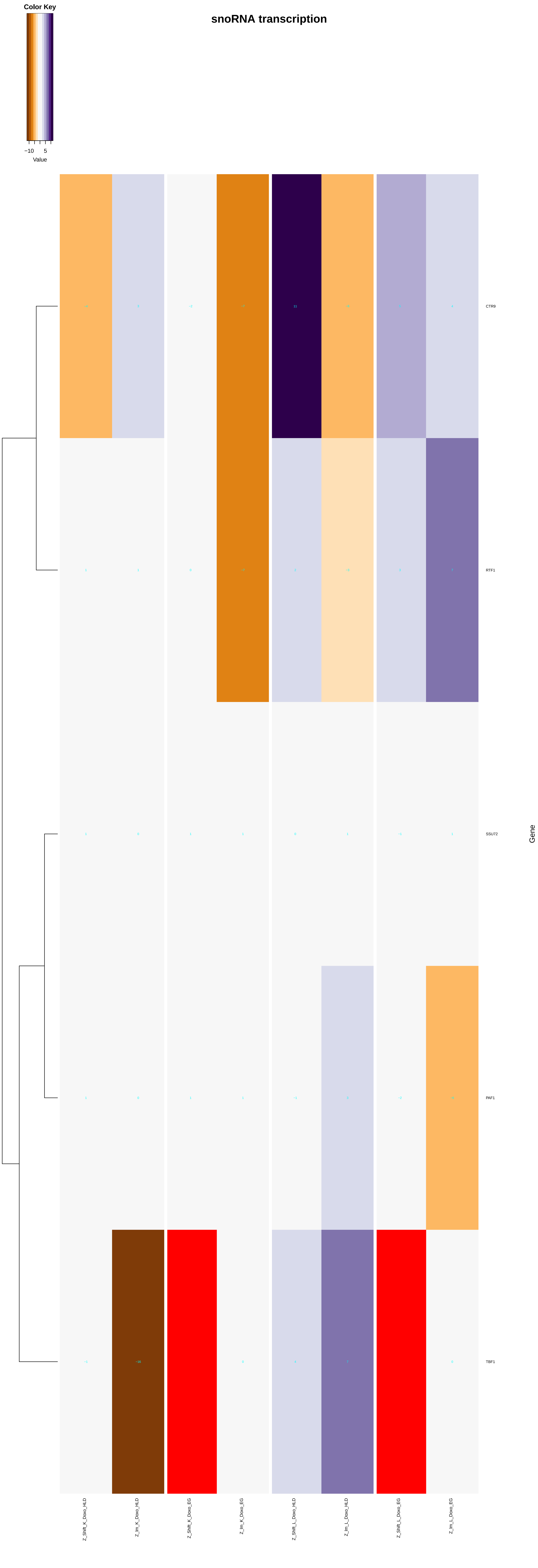
snoRNA transcription
Color Key
−10
5
Value
CTR9
RTF1
SSU72
PAF1
TBF1
Gene
Z_Shift_K_Doxo_HLD
Z_Im_K_Doxo_HLD
Z_Shift_K_Doxo_EG
Z_Im_K_Doxo_EG
Z_Shift_L_Doxo_HLD
Z_Im_L_Doxo_HLD
Z_Shift_L_Doxo_EG
Z_Im_L_Doxo_EG

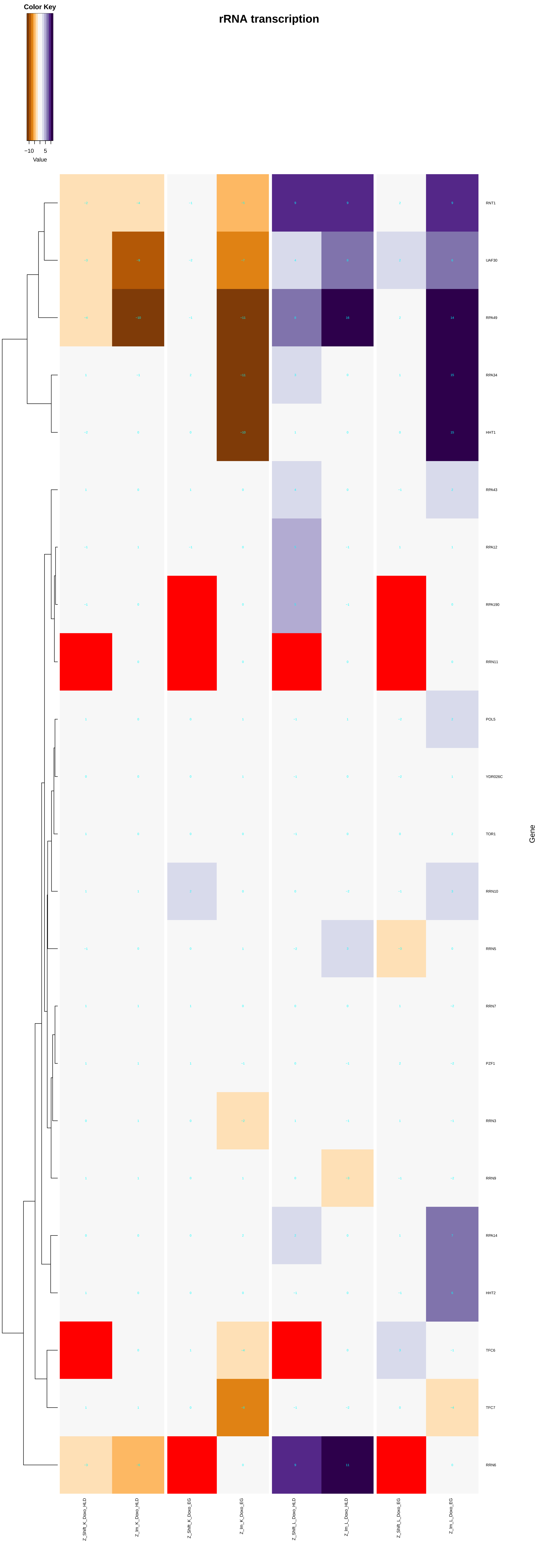
rRNA transcription
Color Key
−10
5
Value
RNT1
UAF30
RPA49
RPA34
HHT1
RPA43
RPA12
RPA190
RRN11
POL5
YDR026C
TOR1
RRN10
RRN5
RRN7
PZF1
RRN3
RRN9
RPA14
HHT2
TFC6
TFC7
RRN6
Gene
Z_Shift_K_Doxo_HLD
Z_Im_K_Doxo_HLD
Z_Shift_K_Doxo_EG
Z_Im_K_Doxo_EG
Z_Shift_L_Doxo_HLD
Z_Im_L_Doxo_HLD
Z_Shift_L_Doxo_EG
Z_Im_L_Doxo_EG

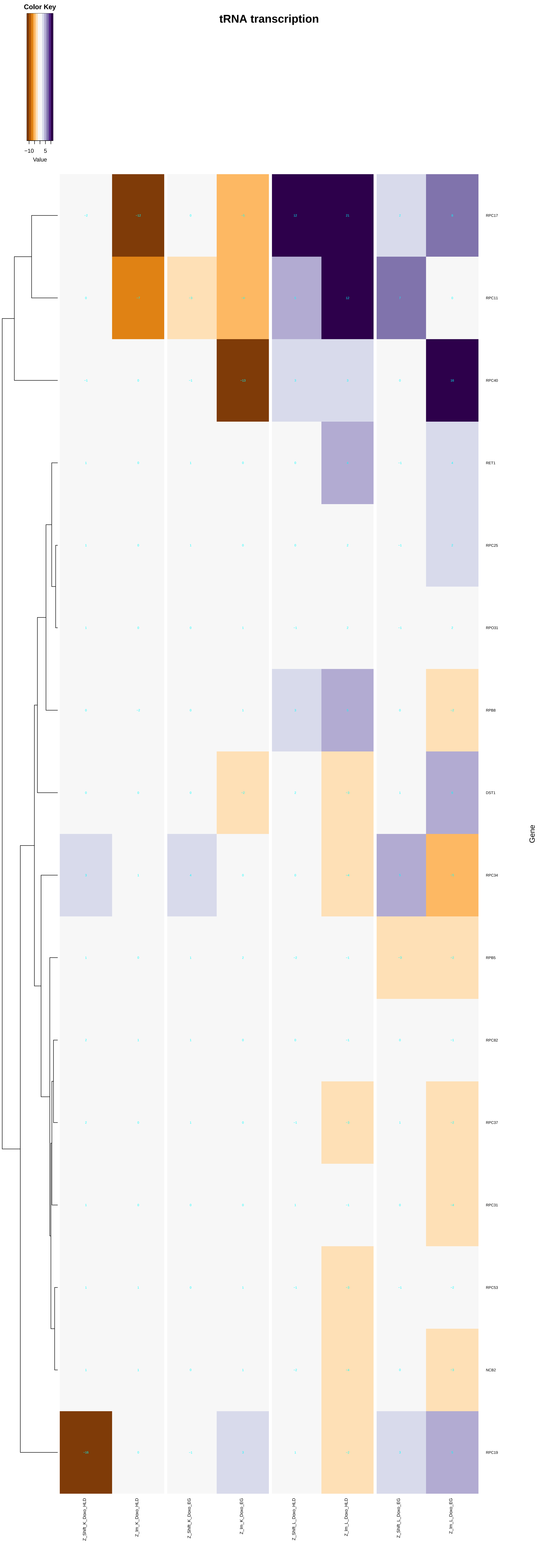

tRNA transcription
Color Key
−10
5
Value
RPC17
RPC11
RPC40
RET1
RPC25
RPO31
RPB8
DST1
RPC34
RPB5
RPC82
RPC37
RPC31
RPC53
NCB2
RPC19
Gene
Z_Shift_K_Doxo_HLD
Z_Im_K_Doxo_HLD
Z_Shift_K_Doxo_EG
Z_Im_K_Doxo_EG
Z_Shift_L_Doxo_HLD
Z_Im_L_Doxo_HLD
Z_Shift_L_Doxo_EG
Z_Im_L_Doxo_EG
